# Barriers to Digital Health Adoption in Older Adults: Scoping Review Informed by Innovation Resistance Theory

**DOI:** 10.2196/75591

**Published:** 2026-02-02

**Authors:** Yosefa Birati, Roy Tzemah-Shahar

**Affiliations:** 1 The Cheryl Spencer Department of Nursing, Faculty of Social Welfare and Health Sciences, University of Haifa, Haifa, Israel, 972 522684065; 2 The Center of Research and Study of Aging, University of Haifa, Haifa, Israel

**Keywords:** older adults, digital health, telemedicine, technology resistance, technology adoption, primary health care, innovation resistance theory

## Abstract

**Background:**

The transformation of digital health technologies has reshaped health care delivery in primary care. Despite these benefits, older adults remain among the most resistant users. Traditional technology adoption models may not fully capture this reluctance, which is shaped not only by usability challenges but also by emotional, psychological, and identity-related concerns. Innovation resistance theory (IRT) offers a complementary framework focused on barriers to adoption rather than solely on facilitators.

**Objective:**

This study aims to map and synthesize evidence on older adults’ resistance to digital health in primary care through the lens of IRT, and to examine how resistance factors align with, extend, or refine IRT’s functional and psychological barriers.

**Methods:**

A scoping review with concept-driven thematic synthesis was conducted. A search for studies published between 2014 and 2025 was conducted across 5 databases: PubMed, CINAHL, Ovid Medline, Web of Science, and Scopus; the final search was completed in November 2025. Eligible studies were those that examined barriers or resistance to digital health use among adults aged 60 years and older in primary care settings. Search terms included “older adults,” “digital health/eHealth,” and “technology resistance.” We excluded studies outside primary care and in which caregivers or health care professionals were the primary users. Data were extracted into a structured matrix and coded to the IRT domains: usage, value, risk, tradition, and image barriers. Relational integration was used to examine co-occurrence and linkages among barriers to inform the conceptual model.

**Results:**

Seventeen studies were included, comprising 6822 participants (sample sizes ranged from 11 to 4525). Most studies were conducted in high-income Western countries, predominantly with qualitative designs, alongside mixed-methods and cross-sectional surveys. Functional barriers included usability challenges, interface complexity, and age-related impairments. Psychological resistance was linked to emotional discomfort, symbolic misalignment, and concerns about the loss of relational care. Value and risk concerns included distrust in diagnostic accuracy, privacy and data security, and skepticism about care quality. Traditional preferences for face-to-face interactions and generational digital divides reinforced image-based resistance. Interactions between barriers were identified, with low self-efficacy and technology anxiety creating feedback loops that reinforce avoidance behaviors.

**Conclusions:**

Older adults’ resistance to digital health is not simply a lack of adoption but a complex, emotionally grounded process involving functional, psychological, and identity-based barriers. This review applies IRT to primary care digital health, shifting the focus from adoption facilitators to resistance mechanisms and integrating co-occurrence patterns into a conceptual model. The synthesis reveals interacting factors of usability, self-efficacy, anxiety, trust, and legitimacy concerns that reinforce avoidance, suggesting that implementation strategies should extend beyond technical usability to rebuild trust, preserve relational care, and align digital solutions with older adults’ values. Review limitations include the predominance of Western-based studies and limited longitudinal data on how resistance evolves.

## Introduction

### Background

The digital transformation of health care has been driven by the integration of telemedicine, mobile health (mHealth) applications, electronic health records, and wearable devices, which have significantly reshaped the delivery of medical services. These innovations address the limitations of traditional care models, which often struggle to meet the evolving demands of health care, particularly for aging populations in rural or underserved areas [1]. By improving access to care, supporting chronic disease management, and promoting preventive health care initiatives, digital health technologies offer promising solutions.

Notably, older adults, who often face mobility limitations, chronic illnesses, and restricted access to traditional health care services, are likely to gain substantial benefits from these digital health innovations [2,3]. However, despite the potential benefits, older adults remain among the most resistant groups to adopting these technologies [4]. This reluctance is widely documented in prior research and often attributed to multiple factors, including limited digital literacy, usability concerns, lower self-efficacy, privacy concerns, and a strong preference for in-person health care interactions. These barriers contribute to older adults’ limited willingness to engage with digital health care solutions [5-7].

The persistence of this reluctance suggests that adoption-centric models may offer an incomplete explanation, highlighting the need for complementary resistance-focused frameworks. To better understand these patterns, we first reviewed established technology adoption models used in health care, clarifying their scope and limitations, and then introduced innovation resistance theory (IRT) as a complementary resistance-focused framework.

### Existing Technology Adoption Models

Established theoretical models, such as the technology acceptance model (TAM) and the Unified Theory of Acceptance and Use of Technology (UTAUT), have been widely used to explain individuals’ adoption and use of new technologies [8]. These models highlight factors such as perceived usefulness, ease of use, performance expectancy, and social influence as key determinants of technology adoption [9,10]. Complementary to these, Rogers’ Diffusion of Innovations Theory describes how new technologies spread through populations by considering factors such as adopters’ characteristics, communication channels, and social systems [11]. These frameworks have been extensively validated and remain central tools for understanding and measuring technology acceptability and usage intentions in health care.

In the context of older adults’ digital health use, adoption-focused models provide valuable insights into factors associated with acceptance and initial uptake; however, prior literature suggests that older adults’ persistent nonuse and resistance are also shaped by affective, psychological, and contextual factors that are not always represented as central constructs in these models [12]. For example, a scoping review by Wilson et al [13] applied UTAUT2 as an analytic framework to map barriers and facilitators to eHealth use among older adults. They identified gaps in the evidence base for certain UTAUT2 constructs (eg, habit and hedonic motivation) alongside recurring concerns related to privacy, trust, and support needs [13]. Another empirical study showed that older adults’ intention to use mHealth was not explained solely by perceived ease of use and perceived usefulness, with person-related, technology-related, and contextual barriers influencing adoption [14]. Fox and Connolly further argue that research on older adults’ resistance to mHealth remains limited and therefore examine how privacy concerns, trust, and risk beliefs influence willingness to adopt beyond standard adoption-model constructs [15]. Taken together, these findings suggest that complementing adoption-focused models with resistance-oriented frameworks may better capture why some older adults actively avoid digital health technologies, including perceived risks, emotional discomfort, and contextual constraints [12,16-18]. Accordingly, adoption-focused models may emphasize intention and perceived benefits, whereas nonadoption can also reflect an active decision-making process shaped by perceived risks and psychological discomfort. Therefore, we propose complementing adoption-focused theories with a resistance-oriented framework, such as IRT.

### Innovation Resistance Theory (IRT) as a Conceptual Framework

IRT, introduced by Ram and Sheth [19], was developed to understand consumer resistance to marketing innovations and their behavior. Unlike models that emphasize adoption facilitators, IRT focuses on understanding why individuals hesitate or actively refuse to adopt new products, services, and ideas, even when they offer potential benefits [19]. The strength of IRT lies in its focus on perceived barriers rather than enablers, making it well-suited for populations such as older adults, where complex emotional, cognitive, and contextual factors influence nonuse. By focusing on the barriers, IRT offers a different perspective that shifts attention from the characteristics of innovations themselves to the reasons behind consumer reluctance to adopt them, especially when such adoption threatens established habits and routines or involves perceived risks [20-22]. In this view, resistance is not merely a lack of adoption but an active process that focuses on barriers to acceptance, including functional, psychological, and social resistance factors [19].

Resistance is defined as a multidimensional construct encompassing 3 dimensions: cognitive resistance, which involves individuals’ appraisal of innovations and their perceived risks; affective resistance, which stems from emotional responses such as fear, frustration, or anxiety; and behavioral resistance, which manifests in actions ranging from passive disengagement to active opposition [23,24]. Within the IRT, these dimensions are further classified into functional and psychological barriers. Functional barriers include the usage barrier, which reflects the extent to which an innovation is perceived as requiring changes to established routines or habits; the value barrier, which arises when the individual perceives that the benefits of an innovation do not outweigh its costs; and the risk barrier, which represents concerns about the financial, functional, and social consequences of adopting an innovation.

Psychological barriers encompass traditional barriers, which refer to the degree to which an innovation forces an individual to accept changes that challenge cultural norms or long-standing behaviors, and image barriers, which relate to the degree to which an innovation is perceived as having an unfavorable image or negative associations [19,25]. These psychological categories often reflect deeper symbolic concerns, such as identity, generational belonging, or perceived legitimacy of digital care. This classification allows IRT to capture the multifaceted nature of resistance in older populations, particularly their emotional unease, normative preferences, and experiential distrust of digital systems. By categorizing resistance into functional and psychological barriers, IRT may provide a comprehensive framework for understanding why older adults struggle to adopt digital health solutions.

Over time, IRT has gained strong empirical support across different service and technology contexts. For example, in mobile banking research across Thailand and Taiwan, IRT barriers explained 60%‐66% of the variance in resistance intentions, with usage, value, risk, and image barriers showing statistically significant effects [21]. In a large Italian survey, Spinelli et al [26] showed that usage barriers and value-related concerns significantly reduced both actual mobile payment use and intention to adopt, whereas risk and image barriers had weaker or nonsignificant effects, and their impact varied across technology-readiness clusters [26]. Similarly, a study of Internet and mobile banking in Finland found that the value barrier was the dominant inhibitor of adoption and intention to adopt, while image and tradition barriers differentiated postponers from rejecters across seemingly similar service innovations [20].

Together, these findings demonstrate that IRT-based barriers have substantial explanatory power for resistance to digital innovations. Therefore, in this review, we apply IRT to structure the evidence on older adults’ resistance toward digital health technologies and to examine whether the identified resistance factors map onto, extend, or refine the original IRT barrier categories. The aim of this scoping review was to synthesize and conceptualize evidence on older adults’ resistance to digital health technologies in primary care using IRT. Specifically, we aimed to identify and categorize resistance factors into IRT functional and psychological barriers and to examine how these barriers co-occur and interact to inform a conceptual model of resistance. The review was guided by the following research questions: (1) What is known from the existing literature about older adults’ resistance to using digital health technologies for monitoring and management in primary health care? (2) What are the functional (usage, value, risk) and psychological (tradition, image) IRT barriers reported across studies? (3) How do IRT barriers co-occur and link within and across studies?

## Methods

### Study Design

The methodology for this scoping review follows the framework proposed by Arksey and O’Malley [27], incorporating refinements by Levac et al [28], and the Joanna Briggs Institute (JBI) Reviewers’ Manual [29]. We selected the scoping review approach to explore the current body of knowledge regarding older adults’ resistance to digital health technologies through the lens of IRT, as it is well-suited to mapping the existing literature, identifying and interpreting patterns of functional and psychological resistance across heterogeneous study types. Within this design, our goal was to provide a theory-informed synthesis that evaluates how well IRT accounts for older adults’ resistance to digital health in primary care and to identify conceptual and empirical gaps that warrant further investigation and measurement development. The reporting of this scoping review was guided by the Preferred Reporting Items for Systematic Reviews and Meta-Analyses Extension for Scoping Reviews (PRISMA-ScR) guidelines [30]. Reporting of the search methods followed the Preferred Reporting Items for Systematic Reviews and Meta-Analyses literature search extension checklist (PRISMA-S) [31] to ensure transparent and complete reporting. The completed PRISMA-ScR checklist is provided in Checklist 2, and the PRISMA-S checklist is provided in Checklist 1.

### Stage 1: Identifying the Research Question

The review was guided by predefined research questions (presented at the end of the Introduction section) informed by IRT and scoping review guidance.

### Stage 2: Searching and Identifying Relevant Studies

A literature search was conducted across 5 major databases: PubMed, CINAHL, Ovid Medline, Web of Science, and Scopus, to identify peer-reviewed publications relevant to the research question. These databases were selected for their broad coverage of health, behavioral, and interdisciplinary studies on older adults and digital health. Each database was searched separately through its web interface, and all retrieved records were exported to Mendeley (version 1.63.0; Mendeley Reference Manager) for deduplication. Review studies were not included in this scoping review; however, their reference lists were screened to identify potentially eligible primary studies. No study registries were searched. Apart from reference-list screening, no additional sources were searched, and no citation searching was undertaken. We did not contact authors to identify additional studies, and no other search methods were used beyond those described. We did not use any previously validated search filters. Search strategies were developed specifically for this scoping review by the authors and were not peer reviewed by an independent expert before execution. We did not adapt or reuse search strategies from previous literature reviews for any substantive part of our search.

The search was carried out on December 20, 2024, and was rerun on November 18, 2025, to identify newly published studies since the initial search. The search followed the JBI PCC structure (Participants, Concept, Context) and combination of the following keywords and MeSH terms: “older adults,” “elderly,” phenomena of “digital health,” “eHealth,” “Telemedicine,” and context of “primary health care,” and “barriers to health technology.” Boolean operators were used to combine search strings (eg, AND, OR). Title and abstract screening and full-text review were conducted by 2 independent reviewers (YB and RTS). The search strategy and keyword combination can be found in Multimedia Appendix 1. Additionally, reference lists of included studies were manually screened to identify relevant studies not captured in the initial searches.

### Stage 3: Selecting the Relevant Studies

#### Inclusion and Exclusion Criteria

The review included papers that met predefined inclusion and exclusion criteria aligned with the JBI PCC framework for scoping reviews (Table 1).

**Table 1.** Study eligibility criteria (Population-Concept-Context) for the scoping review.

Criteria	Inclusion	Exclusion
Participants/population	Older adults aged 60 years and older	Children, adolescents, and younger adults aged <60 years Caregivers Health care professionals
Concept (intervention)	Use of mHealth^a^ for monitoring and management mHealth: telemedicine, mobile phone apps, smartphone apps, web-based systems Resistance or barriers to the use of digital health technologies	Use of mHealth telemonitoring for patients who are not adults and younger adults aged <60 years Use of mHealth telemonitoring by caregivers or health care professionals
Context (cultural factors, geographic location, setting)	Use of digital health technologies in primary health care	Secondary/tertiary care: hospital inpatient wards, surgical centers Emergency/urgent care
Type of studies	Qualitative, quantitative, or mixed methods studies Observational and experimental, cross-sectional, or longitudinal, randomized controlled trial or nonrandomized or noncontrolled trial, case series or case reports	Conference abstracts, editorials, commentaries, letters to editor, essays, book chapters, and books
Language	English	Language other than English
Publication date	From 2014	—^b^

a mHealth: mobile health.

b Not applicable.

The context of the review centered on the resistance to digital health within the framework of IRT. Publications addressing the use of digital health within the resistance domains of usage, value, risk, traditional, and image barriers were considered, while those focusing solely on the description of digital health adoption and facilitators were excluded. Also, no minimum sample size threshold was applied. Consistent with the objectives of a scoping review, studies were eligible regardless of their sample size to maximize coverage of designs (qualitative, quantitative, mixed methods) and contexts.

#### Study Selection Process

The studies were screened against the inclusion and exclusion criteria developed by the authors. The selection process followed three steps: (1) Title and abstract screening to remove irrelevant or duplicate records; (2) Full-text review based on predefined inclusion and exclusion criteria; and (3) Final inclusion based on relevance for examination of resistance to digital health technologies among older adults.

A total of 4976 records were identified through database searches, and 2387 duplicates were removed. After screening 2589 titles and abstracts, 227 full-text articles were reviewed. Two independent reviewers (YB and RTS) evaluated relevant publications for eligibility and selected qualifying publications based on the inclusion/exclusion criteria. We used a consensus-based approach, prioritizing unanimous agreement through re-evaluation of the eligibility criteria; if consensus could not be reached, a third reviewer would adjudicate. An initial pilot screening was conducted independently by both reviewers to ensure consistent interpretation of the eligibility criteria. Discrepancies identified at this stage were resolved through discussion and used to refine the criteria, resulting in full agreement during subsequent screening. A total of 17 studies met the inclusion criteria and were included in the final synthesis. A PRISMA flow diagram illustrates the selection process.

### Stage 4: Charting the Data - Data Extraction and Synthesis

Two authors independently extracted data from all included studies. Data were charted using a standardized extraction form developed for this review, capturing study design, aims, population, type of digital health intervention, and resistance-related findings. Using a concept-driven thematic synthesis, findings were organized into 5 resistance categories from the IRT: usage, value, risk, tradition, and image barriers. A structured matrix was used to map resistance dimensions across the studies. Barrier statements were first open-coded descriptively and then mapped to one IRT family using prespecified rules. Data charting was conducted by the 2 authors, and disagreements were resolved by consensus.

### Stage 5: Collating, Summarizing, and Reporting the Results

Findings were organized in three layers: (1) mapping of the evidence base (study characteristics, settings, modalities), (2) concept-driven qualitative synthesis using IRT classification (usage, value, risk, tradition, and image), and (3) relational integration examined interconnections across IRT barriers. We extracted and coded barrier co-occurrences and linkages reported in the studies’ results sections and participant quotations when two or more barriers were described as co-occurring or interacting. Links were considered explicit when directly stated, inferential when implied within a study’s narrative context, and integrative when consistent patterns recurred across multiple studies (worked examples are provided in the Results).

## Results

### General Characteristics of the Studies

**Figure 1.**
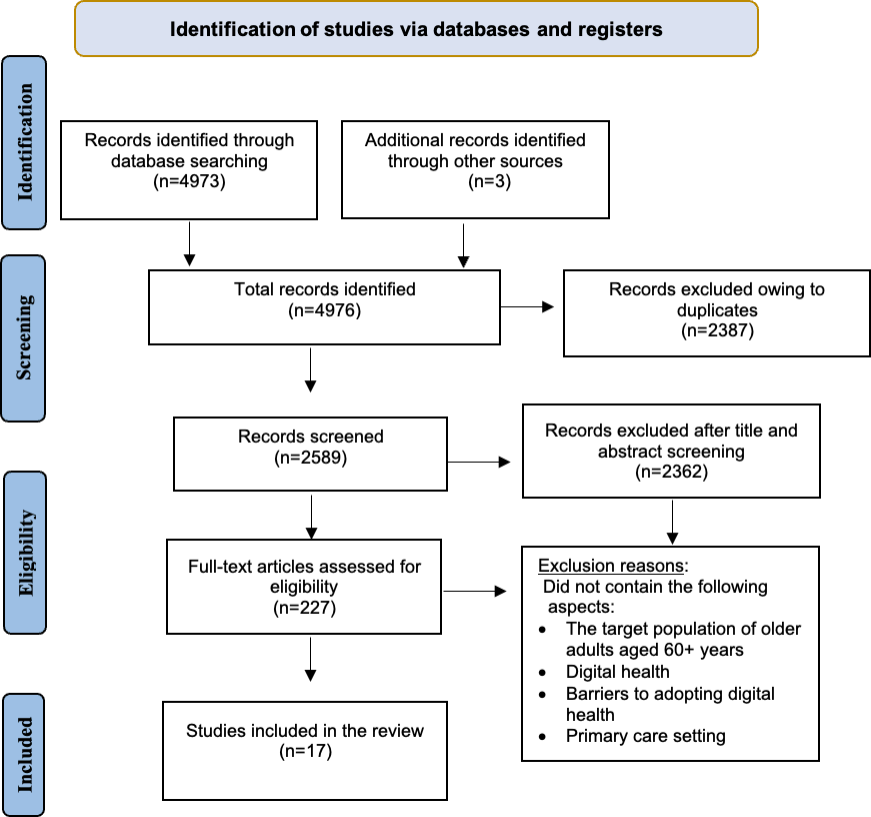
PRISMA (Preferred Reporting Items for Systematic Reviews and Meta-Analyses) flow diagram of the screening and selection process for the Scoping Review on resistance to digital health among older adults.

The database search initially identified 4976 records. After removing duplicates, screening titles and abstracts, and full papers, 17 studies were included in the final synthesis (Figure 1). The included papers represent a predominantly high-income Western countries from the United States (n=4), Sweden (n=3), the Netherlands (n=3), Canada (n=2), Finland (n=1), Norway (n=1), and the United Kingdom (n=1), with only 2 studies from non-Western settings Israel (n=1) and Indonesia (n=1). Eleven studies were qualitative, 3 studies were cross-sectional, and 4 studies were mixed methods designs. Sample sizes ranged from 11 to over 4500 participants, though qualitative samples were generally smaller and in-depth. In terms of digital health modalities, most studies focused on telemedicine or digital consultations (12/17) and patient portals or eHealth services (8/17), with comparatively few studies examining mobile apps or tablets (3/17) and wearables or remote monitoring (2/17) (Table 2).

**Table 2.** Included studies on older adults’ resistance to digital health technologies (n=17). This table presents the country, study design, population sample size, age range, and digital health modality.

	Study design	Aims	Study population	Digital health
Khanassov et al [32] (Canada)	Qualitative study: semistructured interviews and 3 focus groups to explore the experiences of both older adults and health care professionals	How do older adults and health care professionals experience the use of telemedicine? What are the facilitators and barriers to telemedicine use in the care of older adults? What recommendations can enhance telemedicine engagement for older adults and health care professionals	29 older adults and health care professionals (family physicians, nurses, social workers, physiotherapists) Age range 65‐90 years	Telemedicine in primary care
Vergouw et al [33] (Netherlands)	Qualitative study: semistructured and think-aloud interviews	Identify the needs, barriers, and facilitators among community-dwelling older adults (60 years and older) with chronic health conditions in using web-based eHealth applications to support general practice services	19 community-dwelling older adults with at least one chronic condition Mean age 73 (SD 5.3) years	eHealth applications for: e-Consultation Schedule e-Appointment e-Prescription ordering e-Lab results viewing Access to e-File
Knotnerus et al [34](Netherlands)	Qualitative study: semistructured interviews thematic analysis	Investigate the experiences of older patients (65 years and older) who use a digital health platform in general practice Identify barriers and facilitators for using digital health Examine whether a practice’s focus on digital health influences older patients’ choice to become a patient at the practice	18 older patients enrolled in 2 general practices Age range 68‐89 years	Digital health platform for: Communicate with general practitioners Appointment scheduling Order repeat medications
Bhatia et al [35] (United States)	Cross-sectional multimethod study: mixed methods (Quantitative and Qualitative: close and open-ended questions)	Understand older adults’ experience with primary care telemedicine since the COVID-19 pandemic Identify satisfaction levels and technical challenges in telemedicine use Provide policy recommendations for the future of telemedicine services	208 older adults (≥65 y) who had a telemedicine visit within primary care visit Mean age 74.4 (SD 4.4)	Telemedicine (telephone and video visits)
Lam et al [36] (United States)	Cross-sectional study: data from the 2018 National Health and Aging Trends Study (NHATS)	Assess the prevalence of telemedicine unreadiness and how older adults may be left behind in the United States when the migration to telemedicine occurred Identify key barriers preventing the use of video-based telemedicine Examine disparities in telemedicine access based on demographic, socioeconomic, and health-related factors	4525 community-dwelling older adults (≥65 y) in the United States Mean age 79.6 (SD 6.9)	Telemedicine (video and telephone visits)
Nymberg et al [37] (Sweden)	Qualitative research using focus group interviews thematic content analysis	Explore older adults’ beliefs, attitudes, experiences, and expectations regarding eHealth services in primary health care Understand factors influencing adherence to eHealth tools in primary care among elderly patients Identify barriers and facilitators affecting older adults’ engagement with eHealth services	15 elderly patients from 3 primary health care centers in Southern Sweden, selected based on chronic disease status and medication use Age range 65‐80 years	eHealth services and use of the mobile phone for: Contacting the health care system via web Self-monitoring of chronic illnesses Seeking medical information
van Houwelingen et al [38] (Netherlands)	A mixed method triangulation design, including a cross-sectional survey study (quantitative phase) and qualitative observations of older adults performing digital tasks in their daily lives	Understand older adults’ readiness for telehealth, particularly videoconferencing Identify factors influencing their intention to use videoconferencing Examine their capacities and barriers in using digital technology in daily life	256 participants in the survey and 15 older adults aged 65 years or older in the qualitative observations Median (IQR) age=71 (67‐76) years	Telehealth, focused particularly on the use of videoconferencing for health care consultations
Laukka et al [39] (Finland)	Survey study with qualitative inductive content analysis of open-ended questions	Investigate the preferences and needs of older adults regarding the use and development of digital health and social services Understand how digital health and social services can be designed to more effectively meet the needs of older adults	1100 Finnish individuals aged 75 and older Age range 75‐99 years	Telemedicine consultations eHealth services
Rochmawati et al [40] (Indonesia)	Exploratory qualitative study using semistructured interviews, thematic analysis	Explore the acceptance of eHealth technology among older adults in primary care Examine perceptions, attitudes, experiences, and expectations of older people patients regarding eHealth services used in primary care	11 Older adults with chronic conditions (diabetes, hypertension) from a suburban primary health clinic in Indonesia Mean age 66.9 years	Digital health technologies (mobile apps, smartwatches) for health monitoring.
Fjellså et al [41] (Norway)	Explorative qualitative study using semistructured interviews	To explore multimorbid older adults’ experiences with participation and eHealth in care coordination with the support of general practitioners and district nurses	20 older adults with multimorbidity (COPD, heart failure, diabetes, and physical disabilities) receiving primary care services Mean age 82 (range 71‐98) years	Patient portals to share and access information Electronic messaging with general practitioners Schedule appointments Order prescriptions
Mao et al [42] (United States)	Mixed methods needs assessment (cross-sectional survey and qualitative interviews)	Identify barriers to telemedicine video visits among older adults with differing socioeconomic backgrounds and primary spoken languages Understand technological, cognitive, and language-related obstacles to telemedicine use Provide recommendations to improve access and engagement with telemedicine	249 older adults from 2 independent living facilities Mean age 84.6 (SD 6.6) years	Telemedicine visits.
Frishammar et al [43] (Sweden)	Qualitative interviews and process data from a Swedish DHP provider	To investigate adoption and usage barriers of digital health platforms among older adults To understand how to facilitate increased adoption and usage of digital health platforms among the elderly	22 older adults aged ≥65 years, including both users and nonusers of digital health platforms, as well as individuals with experience in digital health development Age range 65‐80 years	Video calls Chats Asynchronous messaging
Haimi et al [44] (Israel)	Qualitative study using semistructured interviews.	To identify the challenges and barriers faced by the senior population when utilizing telemedicine services	14 elderly individuals from a primary health care clinic in Israel Mean age=73 (range 66‐85) years	Telemedicine (phone and video visits) electronic medical records prescription refills Digital referrals Electronic messages with the medical provider
Landgren and Cajander [45] (Sweden)	Qualitative, semistructured interviews.	To identify reasons for nonuse of digital health consultations among elderly in rural areas To describe their attitudes toward technology, and possible challenges and opportunities.	13 participants aged >65 years Mean age 74 years	Digital health consultations delivered by video or chat/phone applications in primary care settings
Ahmed et al [46] (United Kingdom)	Qualitative, focus group study.	To explore the experiences, perceptions, and expectations of older adults from 3 minoritized ethnic group backgrounds regarding digitalized primary care services since the beginning of COVID-19.	27 participants age >65 years Median (IQR) age=69 (66.5‐72.5) years	Telemedicine (phone and video visits) Web-based services: View medical records Schedule appointments Order prescriptions
Ufholz et al [47] (United States)	Cross-sectional survey.	To assess telemedicine preparedness of older primary care patients: internet use, device ownership, prior telemedicine experience, concerns, and perceived barriers	30 community-dwelling adults aged ≥65 Age range 65‐89 years	Telemedicine for primary care (video/online visits)
Sproul et al [48] (Canada)	Cross-sectional survey	To determine what technologies and apps are in current use by older adults, to explore the types of technologies and apps that may be of interest to people in this age group, to explore concerns about technologies, and to examine any age-related differences	266 participants aged ≥60 years 60.2% participants were 60‐74 years and 39.8% participants were 75 years or older	Mobile phones Tablets Health-related apps

The IRT framework was used to guide the coding of extracted findings into the 5 barrier domains (usage, value, risk, tradition, and image).

The findings synthesis is presented in the following sections and summarized in Tables3  4.

**Table 3.** Matrix mapping of innovation resistance theory (IRT) functional and psychological barrier domains (usage, value, risk, tradition, and image) across included studies of older adults’ resistance to digital health in primary care (n=17).

	Functional barriers			Psychological barriers	
	Usage barriers	Value barriers	Risk barriers	Tradition barriers	Image barriers
Khanassov et al [32]	Technical challenges Symptom articulation Technology usability	Informality bias Limited use perception	Diagnostic uncertainty Missed diagnosis concern Technology misuse anxiety	In-person preference	Legitimacy gap Unsuitable for complex care
Vergouw et al [33]	Digital learning curve Technology usability Interface complexity	Limited use perception	Privacy and security concerns Technology misuse anxiety	Symptom articulation In-person preference	Legitimacy gap
Knotnerus et al [34]	Technology usability Interface complexity Symptom articulation Digital learning curve	Limited use perception Disrupted continuity of care	Privacy and security concerns Technology misuse anxiety	In-person preference	Legitimacy gap
Bhatia et al [35]	Symptom articulation Technology usability Cognitive and sensory limitations Digital learning curve	N/A^a^	Missed diagnosis concern Technology misuse anxiety Missed diagnosis concern	In-person preference	Legitimacy gap Unsuitable for complex care
Lam et al [36]	Digital learning curve Symptom articulation	N/A	N/A	In-person preference	N/A
Nymberg et al [37]	Digital learning curve Tech usability. Technology anxiety Physical and sensory impairments	Limited use perception	Privacy and security concerns Technology misuse anxiety	In-person preference Preference for physical documentation	Legitimacy gap Generational digital divide
Houwelingen et al [38]	Digital learning curve Technology anxiety Self-efficacy deficit	N/A	Technology misuse anxiety Privacy and security concerns	N/A	N/A
Laukka et al [39]	Interface complexity Self-efficacy deficit Language and terminology complexity Physical and sensory impairments Technology usability	Limited use perception	Fraud and scam concerns Privacy and security concerns	In-person preference Need for familiarity in care	Generational digital divide Unsuitable for complex care
Rochmawati et al [40]	Self-efficacy deficit Digital learning curve	Limited use perception Informality bias	N/A	In-person preference Need for familiarity in care	N/A
Fjellså et al [41]	Technology usability Interface complexity	Limited use perception Informality bias	Diagnostic uncertainty Missed diagnosis concern Technology misuse anxiety	In-person preference Need for familiarity in care	Generational digital divide
Mao et al [42]	Physical and sensory impairments Digital learning curve technical challenges language barriers Cognitive and sensory impairments Symptom articulation Physical and sensory impairments Technical challenges Technology anxiety Interface complexity	Limited use perception Limited use perception	Diagnostic uncertainty	In-person preference	Unsuitable for complex care
Frishammar et al [43]	Digital learning curve Self-efficacy deficit Technology anxiety	Limited use perception Informality bias	Diagnostic uncertainty Missed diagnosis concern Privacy and security concerns	In-person preference	Unsuitable for complex care Legitimacy gap
Haimi et al [44]	Symptom articulation Technology anxiety Language and terminology complexity Technical challenges Physical and sensory impairments	N/A	Missed diagnosis concern	In-person preference	N/A
Landgren and Cajander [45]	Interface complexity Digital learning curve Self-efficacy deficit Technology anxiety	Limited use perception Informality bias	Diagnostic uncertainty Missed diagnosis concern	In-person preference	Generational digital divide
Ahmed et al [46]	Technology usability Language and terminology complexity Interface complexity	Limited use perception	Diagnostic uncertainty Technology misuse anxiety	In-person preference Need for familiarity in care	N/A
Ufholz et al [47]	N/A	Limited use perception	Privacy and security concerns Diagnostic uncertainty	In-person preference	N/A
Sproul et al [48]	Technology usability	Limited use perception	Privacy and security concerns	N/A	Legitimacy gap

a Not applicable.

**Table 4.** Thematic categorization and definitions of digital health resistance barriers subcategories among older adults.

Category and subcategory	Definition
Usage barriers	
Symptom articulation [32,34-36,42,44,45]	Difficulty in effectively describing symptoms or raising multiple health concerns during telemedicine or digital health interactions, often due to sensory limitations, cognitive strain, or unfamiliarity with web-based communication formats
Technology usability [32-35,37,39,41,44-46,48]	Difficulties interacting with digital health tools due to poor interface design, complex navigation, multi-step login processes, or lack of age-appropriate accessibility features
Digital learning curve [33-38,40,42,43,45]	Challenges individuals face in acquiring, applying, and retaining the skills required to use digital health technologies, often due to limited prior exposure or memory-related difficulties
Interface complexity [33,34,39,41,42,45,46]	Obstacles users encounter when engaging with digital platforms due to poor design elements, confusing navigation, and unclear layouts
Technology anxiety [37,38,42,43,45]	Fear or discomfort experienced when using digital health technologies, often stemming from low confidence, mistrust in one’s digital abilities, or intimidation by unfamiliar systems. This anxiety may lead to hesitation or complete avoidance, driven by concerns about making mistakes that could negatively impact one’s health or care
Physical and sensory impairments [35,37,39,42]	Difficulties in using digital health technologies due to age-related sensory and motor impairments, such as reduced vision, hearing loss, or diminished fine motor control
Self-efficacy deficit [38,40,43,45]	A lack of confidence in one’s ability to successfully use digital health tools or perform required technological tasks, often rooted in limited digital literacy, minimal prior experience, or insufficient training and support
Language and terminology complexity [39,42,44,46]	Difficulty using digital health tools due to complex medical, technical, or bureaucratic language, often compounded by limited proficiency in the language used by the platform
Value barriers	
Informality bias [32,40,41,43,45]	Reluctance to engage with digital health tools based on the perception that they lack legitimacy or necessity in medical care, accompanied by a belief that traditional health care methods are sufficient without digital augmentation
Limited use perception [32-34,37,39-43,45-48]	The belief that digital health tools offer little to no added value compared with traditional care methods, resulting in low motivation to adopt or engage with them
Risk barriers	
Diagnostic uncertainty [32,41-43,45,46]	Concerns about the accuracy and reliability of medical diagnosis due to the absence of physical examination, direct visual assessment, and potential miscommunication, which may increase the risk of medical errors
Missed diagnosis concern [32,35,41,43-45]	Fear that health care providers will miss critical patient information and that essential health issues may be overlooked due to the absence of physical exams, technical distractions, or miscommunication in digital health interactions
Technology misuse anxiety [32-35,37,38,41,46]	Uncertainty or fear about using digital health technologies incorrectly, driven by concerns about user error, system malfunctions, or communication failures that could negatively impact care delivery
Privacy and security concerns [33,34,37-39,43,47,48]	Concerns about the confidentiality, security, and accuracy of personal medical information in digital health care services, driven by fears of data breaches, unauthorized access, and unreliable IT systems
Tradition barrier	
In-person preference [32-37,39-47]	A strong preference for face-to-face health care interactions, rooted in trust in direct communication, perceived importance of physical examinations, and the belief that in-person care offers superior quality
Need for familiarity in care [39-41,46]	Preference for established health care routines and trusted provider relationships over digital health solutions, due to a desire for personalized care, continuity with known providers, and a reluctance to alter traditional in-person interactions
Image barrier	
Legitimacy gap [32-35,37,43,48]	Perception that digital health care is less effective and trustworthy than traditional in-person care, driven by concerns about depersonalization, bureaucratic complexity, and reduced reliability, leading to skepticism about its value and quality
Unsuitable for complex care [32,35,39,42,43,45]	Perception that digital health care services are insufficient for addressing complex medical conditions or cases requiring physical examination, due to concerns about thoroughness, accuracy, and the ability to provide a comprehensive diagnosis and care
Generational digital divide [37,39,41,45]	Perception that digital health care is designed for younger users and is difficult for older adults to adopt, due to differences in familiarity, confidence, and digital literacy

Table 3 details the barriers identified by each study, presenting a matrix that maps each study to the usage, value, risk, tradition, and image barriers. Table 4 defines each barrier subcategory and summarizes how these resistance themes were operationalized across the studies.

### Functional Barriers

In the context of IRT, functional barriers refer to resistance stemming from the practical and objective attributes of the innovation itself, including its required usage, perceived value, and associated risks [19].

#### Usage Barriers

Usage Barriers were the most consistently reported resistance factor, found in 16 studies. Older adults face significant usage barriers to adopting digital health technologies, largely due to technical challenges, usability difficulties, and concerns about quality of care. A central theme across studies was interface complexity. Many participants described digital health platforms as confusing, unintuitive, and poorly designed. Common challenges included unclear layouts, unintuitive menus, and multi-step authentication processes requiring repetitive actions such as logging in, remembering passwords, and uploading medical documents [32-34,39,41,42,45,46]. These features increased cognitive load and made even basic digital interactions feel burdensome and prone to mistakes.

The difficulties were compounded by technology usability issues linked to age-related cognitive and sensory impairments. Older adults with a decline in vision, hearing loss, or memory difficulties and reduced fine motor skills struggled with small font sizes, poor audio quality, poorly structured information, and touchscreen sensitivity, which makes many applications inaccessible without assistance [35,37,42,44,48]. In addition, language and terminology complexity emerged as a significant obstacle. Technical jargon or unfamiliar medical terms often made it difficult for users to interpret instructions or understand the content presented on-screen, particularly among those with limited formal education or health literacy [39,42,44,46].

Another recurring issue was the digital learning curve. Older adults reported limited prior experience with digital health tools or services and found it challenging to adapt to new systems [32,34,39-43,45]. This often led to a self-efficacy deficit where individuals doubted their ability to complete digital health tasks independently. These doubts fueled hesitation and reinforced a sense of digital exclusion, leading to frustrations, avoidance behaviors, and a greater need for support before successfully adopting telemedicine tools [38-40,43,45]. Closely related to this was technology anxiety, the fear of making mistakes or causing harm through improper use, which discouraged many from engaging with telemedicine platforms.

Concerns about system reliability and uncertainty about using digital health care tools make older adults feel less confident in their technical abilities and unprepared [32,33,36,39,40,42,43,45], leading to avoidance behaviors, where they opt not to engage with digital health solutions to minimize the risk of errors [37,38,42].

Beyond usability concerns, preadoption resistance arises from changes in communication dynamics within digital health care. In contrast to traditional face-to-face consultations, which allow patients to express multiple health concerns in a single visit and rely on nonverbal cues, digital health platforms, particularly telemedicine services, alter this dynamic. Studies showed that when older adults use digital health services, they struggle to articulate their symptoms or find it difficult to understand medical terminology or provider explanations [39,45]. As a result, they hesitate to fully communicate medical concerns, whether typing them into digital platforms or discussing multiple health issues during digital visits. This contributes to a perception that digital care is less effective than in-person care [34-36], further discouraging older adults from fully embracing digital health technologies.

#### Value Barriers

Value barriers to adopting digital health solutions among older adults primarily stem from informality bias, the perceived lack of necessity of digital tools, concerns about care quality, and misalignment between the effectiveness of available digital health care services and patient expectations [40,43,45]. While many acknowledge that telemedicine may be appropriate for minor health issues and routine follow-ups, they often do not view it as an adequate substitute for in-person consultations. This limited use perception is particularly strong when it comes to complex conditions that require physical examination or long-term management [32,34,41-43,45,46,48].

Skepticism about the effectiveness of remote consultations is a common concern. Many older adults feel that digital platforms fail to capture nonverbal cues, which are essential for accurate medical assessment and effective patient-provider communication. This concern is particularly pronounced among individuals managing chronic illnesses, who consider ongoing physical evaluations and in-person interactions with health care professionals to be vital components of proper care [33]. Moreover, older adults often emphasize the importance of relational continuity with their health care providers, an aspect they feel is disrupted and compromised in digital health environments. Telemedicine is frequently perceived as impersonal and transactional, lacking the trust and emotional support that typically characterize in-person visits, qualities that many older adults highly value in primary care settings [33,34,37,39]. As a result, some individuals refuse to see their providers outside of traditional clinical settings, which further reinforces resistance to digital health solutions [37,42].

Beyond concerns about quality of care, many older adults also question the necessity of digital health interventions, particularly when the current health care system meets their needs effectively [33,37]. Some dismissed telemedicine as a “solution for a nonexisting problem,” believing that traditional in-person visits provide sufficient care without the added complexity of digital tools [33,37,40]. This skepticism is often exacerbated by low digital literacy or past negative experiences with digital health technology, leading many to view telemedicine and digital health apps as unnecessary, ineffective, or not worth the effort required to learn and adapt [37]. When the perceived benefits of digital health do not outweigh the effort and risks associated with adoption, resistance to these solutions remains strong.

#### Risk Barriers

Risk barriers to digital health adoption among older adults primarily revolve around concerns about diagnostic uncertainty and the potential of missed health issues due to the absence of physical examinations, body language, and other visual cues essential to accurate clinical assessment [32,41-46]. Many older individuals worry that the lack of hands-on evaluation in telemedicine could lead to overlooked symptoms or misinterpretations by health care providers. A prominent concern is technology misuse anxiety, which arises from fear of making errors during digital interactions. Participants described anxiety about technical distractions, errors in digital documentation, incomplete data entry, and uncertainty about whether submitted information, such as messages, forms, or test results, would be properly received and understood by their health care team [41,45,46]. These apprehensions are linked to fears of miscommunication with health care providers, incorrect medical decisions, or overlooked health conditions [32-35,37,40].

Beyond diagnostic concerns, older adults express privacy and security concerns. There is a common distrust of the integrity and security of digital health platforms [33,34,37-40,43,47,48], particularly related to fear that personal health data could be exposed to unauthorized access, fraud, or misuse. Some participants described concerns about scams that mimic legitimate digital services, increasing their reluctance to trust or engage with digital health tools [39]. This skepticism is further compounded by uncertainty around how health care institutions collect, store, and share data through electronic health records and patient portals [39].

Additionally, lack of confidence in digital skills was repeatedly cited as a major factor behind misuse anxiety. Older adults often lack confidence in their digital skills, particularly in navigating complex interfaces or troubleshooting technical issues. Common fears included accidentally deleting important information, misunderstanding medical results, or failing to complete critical health care tasks [34,40]. As a result, many preferred to avoid digital health services entirely rather than risk making mistakes that could negatively impact their care.

Another key source of resistance is the perceived loss of autonomy in health care decision-making. Some older adults expressed concerns that eHealth solutions shift decision-making control from patients to automated systems, reducing their ability to advocate for personalized care and communicate effectively with health care providers about their health care [37,41]. This fear is particularly prevalent among those unfamiliar with electronic health records or unaware of how to use digital clinical discussions.

### Psychological Barriers

Psychological barriers refer to resistance stemming from subjective, cognitive, and emotional conflicts between the innovation and the individual’s established traditions and self-image barriers [19].

#### Tradition Barriers

Traditional barriers to digital health adoption among older adults arise from long-established care routines, personal preferences for in-person interactions, and a strong need for familiarity in health care interactions. Many older adults have their health-seeking behaviors around face-to-face consultations, expressing satisfaction with traditional care models and questioning the necessity or value of digital alternatives [37,40,41]. They often perceive little incentive to switch to eHealth services when current systems already meet their expectations [37,44,45]. A central theme is the belief that *in-person interactions* offer superior quality of care, stronger provider-patient relationships, and greater emotional warmth. Digital platforms are often seen as impersonal, lacking the human touch and nonverbal communication cues that older adults consider essential for effective medical consultations [32,34-36,39,41,43,45-47]. This is especially concerning for individuals managing chronic conditions or complex health issues, where verbal-only communication may be insufficient for accurate symptom reporting and clinical assessment [32,33].

The need for familiarity in care also contributes to resistance toward digital health adoption. Many older adults prefer continuity with known health care professionals, such as physicians, nurses, or other health care professionals, and value personalized guidance and documentation, such as printed instructions or handwritten over generic digital content. Some do not want all services to be transferred through digital platforms, especially when health care and social service issues are too complex to be handled without face-to-face contact [35,37,39,42,46].

Another common concern is the perceived legitimacy of telemedicine. Some older adults do not view phone or video consultations as valid medical encounters, describing them as informal and lacking the authority of traditional office visits [32]. This perception is heightened among individuals who had not used digital health before the COVID-19 pandemic and who experienced the rapid shift to telehealth as both disruptive and disorienting, owing to complex interfaces and limited user guidance [34]. For these individuals, digital health solutions interfere with familiar health care routines and pose significant adaptation challenges [39].

#### Image Barriers

Image barriers to digital health adoption among older adults arise from negative perceptions of technology, distrust in digital health solutions, and skepticism about their legitimacy and effectiveness in clinical care. Many older adults associate digital health technologies with lower quality of care and consider them as an unacceptable alternative to traditional in-person visits [35,42]. For some, these technologies are viewed as overly complex, impersonal, and rigid, contributing to a Legitimacy Gap, a perception that digital health care lacks the authenticity, reliability, and interpersonal value of conventional medical interactions [33,43,48]. This skepticism is reinforced by the belief that health care should be hands-on, personalized, and relational, the qualities they feel digital platforms fail to deliver.

Another central issue underlying this perception is the Generational Digital Divide. Many older adults view digital health tools as designed primarily for younger, digitally proficient users, and they report feeling excluded or disadvantaged by their limited experience with digital technologies [37,39-41,45]. This belief is often coupled with self-perceived technological inadequacy, where individuals feel “too old” to learn or incapable of using new systems effectively [39]. These psychological barriers are compounded by negative past encounters with health care bureaucracy or poorly designed interfaces, which foster the impression that digital health prioritizes efficiency over patient-centered care [37]. Additionally, difficulties navigating eHealth platforms often lead to a sense of powerlessness in managing their health, further alienating them from digital solutions.

Older adults also view telemedicine and digital health as unsuitable for both routine and complex care needs [32,42,43]. Many perceive these technologies as inferior to traditional, in-person medical consultations, citing concerns about their inability to provide thorough physical examinations, comprehensive assessments, and hands-on diagnostics [39]. Digital health is also associated with social isolation and reduced autonomy, as some fear that shifting toward digital health care may limit direct patient-provider interactions and diminish their role in medical decision-making [34]. This contributes to a strong preference for traditional care models, where in-person visits provide greater trust, familiarity, and perceived quality.

### Evidence and Gap Map

**Figure 2.**
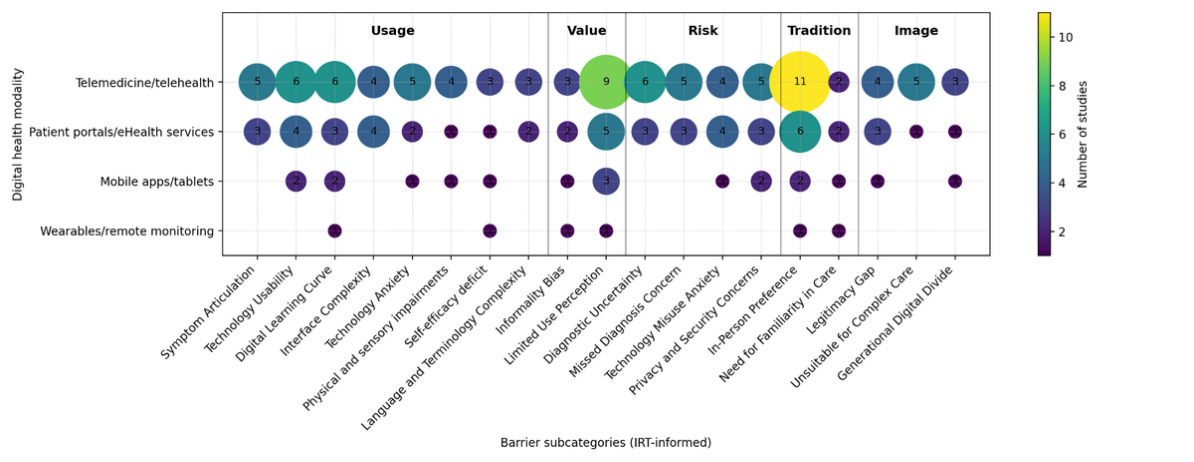
Evidence and gap map of digital health modalities by IRT-informed resistance subcategories in primary care among older adults. Bubble size and color intensity represent the number of included studies contributing to each intersection (n=17). IRT: innovation resistance theory.

Across the included studies, there was substantial variation in both the types of digital health technologies examined and the specific resistance factors reported. To strengthen the mapping component of this scoping review, we developed an evidence and gap map to summarize the distribution of evidence and identify gaps across digital health modalities and resistance constructs. Guided by IRT, we categorized studies by the type of digital health modality and by IRT-informed barrier subcategories derived from the extracted findings (Figure 2).

Specifically, the map highlights that evidence is concentrated in studies of telemedicine and patient portals or eHealth services, with fewer studies addressing mobile apps or tablets and minimal evidence on wearables or remote monitoring. Across modalities, frequently represented barriers included usability and interface complexity, self-efficacy and technology anxiety, and trust-related concerns such as privacy, data security, and perceived legitimacy of digital encounters. In contrast, several modalities-barrier intersections show limited or absent evidence, indicating that resistance to certain technologies, particularly wearables and app-based monitoring, remains underexplored in primary care contexts.

### Conceptual Integration: Interconnected Barriers Leading to Digital Health Avoidance

Across the 17 included studies, usage barriers were the most consistently reported (16/17 studies). Risk barriers and tradition barriers were also prevalent (15/17 studies). Value barriers were common (13/17 studies), and image barriers were reported in a smaller, but still substantial subset (11/17 studies). Co-occurrence patterns were apparent across domains, and worked examples illustrate how linkages were derived. For example, one participant described limiting use to familiar functions and avoiding other features, indicating a usage barrier, accompanied by anxiety when stepping outside her comfort zone, suggesting an affective risk component and fear of making mistakes: “I never look over there, I just do everything I have learned… Outside of that, I become nervous.” [38]. In another study, a participant noted that he did not grow up with technology, indicating a usage barrier related to limited digital familiarity, and expressed a tradition barrier by preferring to arrange appointments by phone and speak with the physician face-to-face rather than use digital channels: “But we did not grow up with the computer. I would rather make a phone call to arrange an appointment and prefer to talk face-to-face to the physician” [33]. Another participant questioned the adequacy of digital encounters for a proper clinical assessment, reflecting an image or quality concern that co-occurred with a tradition-related preference for face-to-face care and an implied need for greater diagnostic assurance (risk): “I would rather that the doctor can actually touch me, examine me with a stethoscope… I also think in-person communication is sometimes better…” [42]. Together, these patterns suggest that resistance is rarely attributable to a single factor; rather, studies frequently report clusters of functional and psychological barriers that co-occur. These recurring clusters informed the relational integration step; linkages were coded as explicit when directly stated in study results or participant quotes, inferential when implied through within-study co-occurrence and narrative context, and integrative when synthesized across multiple studies showing consistent patterns.

**Figure 3.**
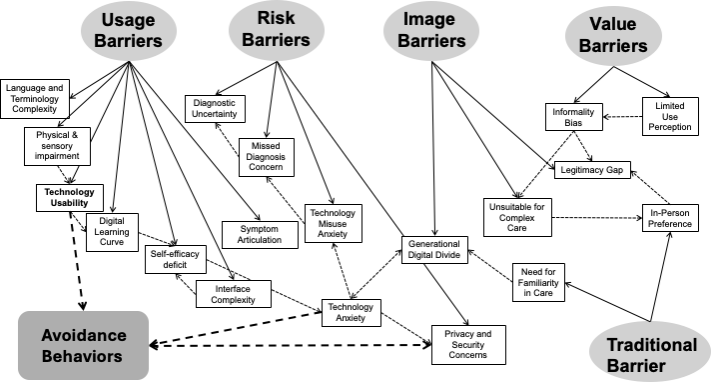
Conceptual model of interconnected resistance barriers leading to digital health avoidance among older adults in primary care, interacting co-occurrence patterns across included studies to illustrate directional relationships and feedback loops.

As part of the relational integration step of our synthesis (Stage 5), we developed a conceptual model that integrates the identified barriers into an interconnected structure (Figure 3). This conceptual integration was undertaken to move beyond listing individual barriers and to summarize recurring co-occurrence patterns observed across the studies. The interconnected nature of resistance barriers creates a self-reinforcing reaction cycle that leads to avoidance behaviors among older adults. Rather than operating in isolation, functional and psychological barriers interact dynamically, compounding resistance and entrenching disengagement from digital health platforms.

Technology usability challenges contribute to difficulties in the digital learning curve, which, along with interface complexity, results in a self-efficacy deficit and a lack of confidence in using digital health technologies. This diminished self-efficacy further fuels technology anxiety, increasing hesitation and discouraging engagement. Importantly, these usability issues do not just reduce confidence; they initiate a cascade of psychological barriers that elevate emotional discomfort and cognitive overload. Figure 3 illustrates this cascading effect: a feedback system where usability problems initiate low self-efficacy, which in turn escalates into technology anxiety. This psychological discomfort amplifies risk perceptions, including fear of misdiagnosis, privacy breaches, and technology misuse. These concerns reduce trust in digital health care solutions and reinforce avoidance behaviors. Privacy and security concerns and technology anxiety reinforce each other, creating a cycle of distrust. As the trust in the system diminishes, older adults become less likely to interact with digital platforms, which limits exposure and impedes skill acquisition, further deepening their self-efficacy deficit. This cycle in Figure 3 is illustrated through closed feedback loops, where arrows between barriers represent how one resistance factor amplifies another (eg, Interface Complexity → Low Self-Efficacy → Technology Anxiety → Avoidance).

Traditional barriers, such as a strong preference for in-person care and the need for familiarity, also strengthen image barriers, including the legitimacy gap and the generational digital divide, further discouraging digital health adoption. As shown in Figure 3, these values-based preferences and generational perceptions reinforce internal skepticism with digital tools, particularly when technology is perceived as impersonal. The legitimacy gap reflects older adults’ perception that digital tools lack the authenticity and authority of face-to-face care, while the generational divide reinforces feelings of exclusion from technologies perceived as designed for younger users. Figure 3 also highlights this convergence between identity-based resistance (eg, tradition/image) and capability-based resistance (eg, usability, anxiety). Together, these interrelated barriers form a self-reinforcing loop, where initial usability difficulties and emotional skepticism amplify resistance, which leads to withdrawal from digital health use entirely (Figure 3).

## Discussion

### Principal Findings

This scoping review applied the IRT to examine older adults’ resistance to digital health technologies within primary care contexts. Across the included studies, we found consistent functional barriers (such as usability difficulties, interface complexity, and sensory or cognitive limitations) and recurrent psychological barriers (such as a preference for in-person care and concerns about the legitimacy of digital encounters), with value-related concerns (limited perceived benefit) and risk-related concerns (diagnostic uncertainty, privacy, and security worries) also prominent.

The findings suggest that resistance is not a static failure to adopt nor a passive disengagement, but rather a dynamic, emotionally embedded process. This process is shaped by the interaction of functional and psychological factors, including identity and value-related concerns, which do not operate in isolation but reinforce each other in feedback loops that entrench avoidance behaviors over time. The interplay between usability challenges, emotional discomfort, and value-based misalignment reflects the multifaceted nature of resistance in this population. Also, interrelationships indicated that capability-related barriers erode confidence and increase anxiety, while identity-related concerns reinforce distrust and preference for face-to-face care, together discouraging engagement. Linkages were categorized by evidentiary basis (explicit, inferential, integrative), supporting IRT as a useful framework for organizing and interpreting resistance patterns.

Functional barriers such as interface complexity, digital learning curves, and age-related sensory or cognitive limitations were among the most identified sources of resistance. However, their significance lies not only in their prevalence but in their role as catalysts: they often trigger negative psychological responses, including diminished self-efficacy, anxiety, and fear of error. These emotional reactions contributed to a broader sense of technological vulnerability and led to sustained disengagement, demonstrating how technical design and user experience are deeply interconnected.

Beyond usability, resistance was often rooted in symbolic and identity-related concerns. A preference for face-to-face interactions, generational beliefs regarding technology, and the desire for continuity with known providers were consistently linked to what can be described as symbolic distancing, a form of resistance grounded in perceived legitimacy and personal norms. Even where functionality improved, older adults continued to express skepticism, viewing digital tools as impersonal, exclusionary, or inappropriate for managing complex health needs. This suggests that emotional and symbolic dimensions may play a stronger influence on resistance than previously recognized.

These insights align with earlier theoretical work that repositions resistance as a dynamic, emotionally driven response process. The findings support an evolving theoretical perspective that frames resistance as an active process. Rather than being the inverse of adoption, resistance emerges from distinct cognitive and emotional pathways and may dominate decision-making even in the presence of positive attitudes [49]. Other research has also shown that tradition and identity-based concerns frequently outweigh usability considerations in shaping innovation rejection, particularly in service-oriented settings [20]. This review affirms that older adults’ resistance is rarely due to a lack of awareness or rational evaluation alone, but rather reflects deeply embedded emotional and symbolic stances.

Breaking these loops requires targeted interventions that not only simplify interface design but also rebuild self-efficacy, trust, and the perceived legitimacy of digital care. Accordingly, programs should pair practical usability supports (eg, task simplification, assisted-digital options, scaffolded practice) with psychological strategies (eg, anxiety reduction, trust-building, culturally and linguistically responsive framing).

### Comparison to Prior Work

The findings of this review both align with and challenge established models of technology acceptance. For instance, it complements the critiques of the extended UTAUT, which has been applied to prior studies involving older adults in health care settings. One study has highlighted effort expectancy, perceived usefulness, and trust in health care providers as primary predictors of adoption. While these factors remain relevant, this review suggests they are insufficient to fully account for persistent resistance observed in older populations. This resistance appears to stem not from a lack of understanding but from deeper emotional and symbolic misalignments between digital tools and the users’ personal values, care routines, or generational identities [50]. In this context, resistance is not a knowledge deficit but a deliberate, emotionally grounded response to perceived risks, impersonality, or social exclusion. Our synthesis clarifies how such misalignments link to concrete pathways (eg, usability → low self-efficacy → anxiety → avoidance), adding a mechanism to prior critiques.

Reinhardt et al [51] claim in their study that resistance to innovation is not merely the opposite of adoption but a distinct phenomenon that operates through its own logic and dynamics, and thus warrants a separate theoretical approach. They proposed the concept of “adoption triggers,” external events or contextual changes that interrupt entrenched resistance and enable eventual uptake. This finding aligns with the results of this review, where participants continued to resist engagement even after usability improvements, suggesting that design enhancements alone are insufficient [51]. Psychosocial catalysts such as trust in providers, alignment with identity, or significant life transitions may be necessary to shift deeply embedded resistance patterns.

Further support comes from the argument that TAM and UTAUT, widely used models, were not originally developed for health care but rather in organizational contexts. Like IRT, they were formulated outside the health domain and may require adaptation when applied in complex settings, such as digital health for older adults. In their original formulations, these models assume that perceived usefulness and ease of use directly predict technology acceptance. However, in health care, these assumptions are challenged, especially in the context of older adult users [52]. Health care studies often have to add context-specific variables such as computer anxiety, trust, or physician endorsement to increase explanatory power. This suggests that existing models may benefit from complementary perspectives that foreground resistance shaped by emotional discomfort and identity-related concerns, including symbolic dissonance around how digital health fits with older adults’ roles and expectations. This review affirms the need to view resistance among older adults as socially embedded and identity-relevant, rather than reducible to issues of usability or cognitive evaluation.

Resistance constructs are not intended to replace established acceptance models such as TAM and UTAUT, but to extend them and provide a more complete account of older adults’ technology use and nonuse patterns. Yu et al [53], in their research, also extend UTAUT with aging-specific variables such as perceived physical condition, self-actualization needs, and technology anxiety. Their empirical study among Chinese older adults found that while traditional UTAUT predictors (eg, performance and effort expectancy) remain significant, behavioral use was also shaped by perceived physical limitations and psychological needs for self-fulfillment. Notably, the effect of technology anxiety was nonsignificant, suggesting that usability alone does not explain resistance; rather, broader psychosocial and experiential factors must be considered [53]. These adaptations have introduced constructs such as perceived physical condition, self-actualization needs, and psychosocial well-being to better explain behavioral engagement with health care conversational agents among older adults. Our mapping complements these extensions by locating these constructs within the IRT domains and by indicating which inter-barrier links are explicitly supported by the literature.

### Theoretical Implications

This review advances theory on digital health adoption and resistance among older adults in 2 main ways. First, it refines IRT for the context of aging and digital health by highlighting aging-specific resistance themes such as legitimacy gaps, generational digital divides, and anxiety about technology misuse as candidates for further conceptualization and measurement within the original IRT domains. Second, it points to resistance as a dynamic process in which these barriers interact in feedback patterns rather than operating as isolated categories. This mechanism-oriented view complements existing TAMs by underscoring that persistent nonuse reflects active, emotionally and symbolically shaped resistance, rather than merely weak adoption intentions.

### Practical Implications

From a gerontechnology and age-inclusive design perspective, the IRT-based model translates the identified barriers and linkages into actionable design and implementation levers to reduce resistance among older adults in primary care. This review has important implications for digital health design, practice, and policy.

First, the disproportionate concentration of extant research within high-income Western countries necessitates a nuanced approach to global implementation, as resistance profiles are not homogenous but are contingent upon divergent socioeconomic structures, varying levels of digital literacy, and culture-specific perceptions of aging [54]. Addressing these complexities requires a paradigm shift from a reactive model, characterized by a narrow focus on technical troubleshooting and interface simplification, toward a proactive design. While mitigating interface complexity and accommodating sensory impairments remain fundamental requirements, such technical refinements in isolation are insufficient to resolve resistance that is fundamentally anchored in emotional and psychological factors. Consequently, proactive age-tech development should prioritize the alignment of digital interventions with users’ long-standing traditions and the preservation of relational continuity in care [55]. By acknowledging traditional barriers and framing digital tools as seamless extensions of familiar, trusted care routines rather than disruptive innovations, developers can transition from delivering impersonal technical products to co-creating solutions that resonate with the core identities and values of older populations.

Building on the conceptual model in Figure 2, breaking the self-reinforcing cycle of resistance requires targeted interventions that address both practical usability barriers and underlying psychological resistance; focusing on interface design or digital literacy alone is unlikely to change deeply rooted patterns of nonuse. Designers need to focus not only on functionality but also on providing emotional reassurance and strengthening the perceived legitimacy and social meaning of digital care. Therefore, solutions should be co-designed with older adults not only to ensure they fit with their routines, communication styles, and cultural values, but also to directly address the specific IRT barriers identified in this review by incorporating strategies that reduce friction and promote confidence. These strategies may include simplifying high-friction tasks by using shorter flows, fewer required fields, larger tap targets, and accessible defaults. Also, designers can provide stepwise guidance and “practice mode,” and offer assisted-digital options such as telephone call-back support, shared on-screen navigation with staff, and on-site digital stations within clinics where staff can help patients complete digital tasks.

Privacy, risk perceptions, and distrust emerged as central barriers in our synthesis. Digital health platforms should incorporate trust-enhancing features, including sustained relationships with known providers, easy access to human support, and clear, simple explanations of data practices. To strengthen perceived legitimacy, systems should preserve care delivery choice (seamless switch to phone or in-person visits), display continuity cues (named clinician, photo, prior encounters), and surface concrete benefits (time saved, refill accuracy, faster appointments). Culturally and linguistically responsive content, combined with feedback that reinforces mastery, can further mitigate anxiety and improve self-efficacy, helping to disrupt the self-reinforcing loops that lead to avoidance. Together, these design-oriented recommendations translate our conceptual findings into practical guidance for technology designers and implementers seeking to reduce resistance among older adults.

### Future Research Directions

Future research should investigate the temporal evolution of resistance, including how initial avoidance may shift or diminish over time, and under what conditions. There is a need to explore resistance dynamics among underrepresented populations, such as ethnic minorities, linguistically diverse groups, and individuals living in lower-resource settings. In line with Bevilacqua et al [56], emerging work on service-specific acceptance measures for older adults who developed the Robot-Era Inventory as a tailored acceptance scale for a social robotics platform, and called for customizable, context-specific tools tailored to specific technologies and services for older adults, future studies should develop and validate IRT-informed scales tailored to particular digital health modalities [56]. In addition, longitudinal and mixed-methods designs could provide deeper insight into how resistance is maintained or disrupted. Finally, the development and empirical testing of interventions grounded in IRT would help bridge the gap between theory and design strategies.

### Strengths and Limitations

A key strength of this review is its structured, theory-driven synthesis across diverse empirical studies. By applying the IRT to various study designs and health care contexts, this review enhances the conceptual understanding of digital resistance among older adults. It was conducted according to best-practice guidelines for scoping reviews, which reflect established methodological standards.

Several limitations should be noted. First, the search was restricted to English-language publications, which might have excluded relevant studies published in other languages. Second, the review encompasses studies published between 2014 and 2025, a period characterized by rapid technological advancement. Improvements in device usability during this time may have influenced user experiences and patterns of resistance, potentially affecting cross-study comparability. Third, most of the included studies were conducted in high-income Western countries, and the patterns of resistance identified here may not fully capture experiences in lower-income or non-Western contexts, where digital infrastructures, health systems, and cultural norms around aging and technology may differ substantially. This concentration substantially reduces generalizability beyond high-income Western settings and limits the applicability of our findings to global contexts where digital literacy, socioeconomic factors, and cultural perceptions of aging and health care may create distinct resistance profiles. Fourth, none of the included studies reported participants’ cognitive status or used standardized cognitive screening measures. As a result, we could not examine whether resistance barriers vary by cognitive integrity or distinguish attitudinal resistance from barriers related to cognitive impairment, which may influence learnability, confidence, and sustained use of digital health technologies. Finally, the proposed conceptual model has not yet been validated in practice and should be regarded as hypothesis-generating. Future research should operationalize the IRT domains and evaluate their factor structure, reliability, and predictive validity in empirical studies.

### Conclusions

Applying IRT to older adults’ experiences with digital health shifts the focus from “lack of readiness” or skills gaps to resistance mechanisms and how technologies are designed and integrated into primary care. Resistance emerges as an active, emotionally rooted process involving functional, psychological, and identity-based barriers to adoption, and this review integrates recurring co-occurrence patterns into a conceptual model, thereby moving beyond prior work that lists barriers in isolation. The synthesis clarifies how usability problems can undermine self-efficacy, increase technology anxiety, and amplify trust and legitimacy concerns, creating feedback loops that reinforce avoidance. Real-world implications: implementation strategies should go beyond technical usability by rebuilding emotional trust, supporting relational continuity, and aligning digital solutions with older adults’ values and routines through meaningful channel choice and transparent communication about risks. In addition, IRT offers a structure for developing domain-specific measures and interventions that address usage, value, risk, tradition, and image barriers, supporting a more realistic and equitable digital transformation in primary care for aging populations.

## Supplementary material

10.2196/75591 Multimedia Appendix 1 Online search strategy.

10.2196/75591 Checklist 1 PRISMA-S Checklist.

10.2196/75591 Checklist 2 Preferred Reporting Items for Systematic reviews and Meta-Analyses extension for Scoping Reviews (PRISMA-ScR) Checklist.

## Abbreviations:

IRT: innovation resistance theory
JBI: Joanna Briggs Institute
mHealth: mobile health
PRISMA-S: Preferred Reporting Items for Systematic Reviews and Meta-Analyses literature search extension checklist
PRISMA-ScR: Preferred Reporting Items for Systematic Reviews and Meta-Analyses Extension for Scoping Reviews
TAM: technology acceptance model
UTAUT: Unified Theory of Acceptance and Use of Technology
