# Supplementary material for: Barriers to Digital Health Adoption in Older Adults: Scoping Review Informed by Innovation Resistance Theory
Source: J Med Internet Res. 2026 Feb 2;28:e75591. doi: 10.2196/75591 (PMC12863245; doi:10.2196/75591)
Supplement: Multimedia Appendix 1 [file jmir-v28-e75591-s001.docx]

**Multimedia Appendix 2**. Online search strategy

| Pubmed keywords | ~~#~~ |
| --- | --- |
| "Telemedicine"[MeSH] OR "Mobile Applications" [Mesh] OR "Digital Health"[Title/Abstract] OR telemedicine [Title/Abstract] OR "mHealth"[Title/Abstract] OR "eHealth"[Title/Abstract] OR "mobile health" [Title/Abstract] OR "Remote Consultation"[MeSH Terms] OR "Telehealth"[Title/Abstract] OR "digital technolog*"[Title/Abstract] | 1 |
| "Aged"[MeSH] OR "Older Adults"[Title/Abstract] OR "elder*"[Title/Abstract] OR "Elderly"[Title/Abstract] OR "Geriatric Patients"[Title/Abstract] OR "senior*"[Title/Abstract] | 2 |
| "Primary Health Care"[MeSH] OR "Family Practice"[MeSH] OR "General Practice"[MeSH] OR "Primary care"[Title/Abstract] OR "Primary Health Care"[Title/Abstract] OR "family medicine"[Title/Abstract] OR "General Practice"[Title/Abstract] | 3 |
| #1 AND #2 AND #3 | ~~4~~ |
| Filters: English, from 2014/1/1 – 2025/11/28 | * |

| Medline keywords | # |
| --- | --- |
| telehealth or telemedicine or telemonitoring or telepractice or telecare or Remote Consultation or Digital Health or mHealth or eHealth | S1 |
| elderly or aged or older or elder or geriatric or elderly people or old people or old people or senior or older adults | S2 |
| primary care or primary health care or primary healthcare or general practice or gp or family practice or family medicine | S3 |
| S1 AND S2 AND S3 | S4 |

| Web of science keywords | # |
| --- | --- |
| TS =("Telemedicine" OR "Digital Health" OR "Digital Technolog*" OR "mHealth" **OR "mobile application*"** OR "eHealth" OR "Remote Consultation" OR "Telehealth" (Topic)) and **English** (Languages) and 2025 or 2024 or 2023 or 2022 or 2020 or 2019 or 2018 or **2017** or **2016** or **2015** or **2014** (Publication Years) | 1 |
| **TS=(**"Aged" OR "Older Adults" OR "Older people" OR "Elderly" OR "Geriatric Patients" (Topic)) | 2 |
| **TS=(**primary care or primary health care or primary healthcare or general practice or gp or family practice or family medicine) | 3 |
| **#1 AND #2 AND #3** | 4 |

| CINAHL keywords | # |
| --- | --- |
| MH "Telemedicine" OR MH "Remote Consultation" OR TI "Digital Health" OR TI "mHealth" OR TI "eHealth" OR TI "Telehealth" OR "mobile health" OR AB "Digital Health" OR AB "mHealth" OR AB "eHealth" OR AB "Telehealth" | 1 |
| MH "Aged" OR TI "Older Adults" OR AB "older adult*" OR TI "Elder*" OR TI "Geriatric Patients" OR AB "Older Adults" OR AB "Elderly" OR AB "Geriatric*" OR TI senior* OR AB senior* | 2 |
| MH "Primary Health Care" OR MH "Family Practice" OR MH "General Practice" OR TI "Primary care" OR TI "Primary Health Care" OR TI "General Practice" OR TI "family medicine" OR AB "Primary care" OR AB "Primary Health Care" OR AB "General Practice" | 3 |
| **#1 AND #2 AND #3** | 4 |

| Scopus keywords | # |
| --- | --- |
| (TITLE-ABS-KEY("Telemedicine" OR "Remote Consultation" OR "Digital Health" OR "mHealth" OR "eHealth" OR "Telehealth" OR "mobile health" OR "Digital Health")) | 1 |
| (TITLE-ABS-KEY("Aged" OR "Older Adults" OR "Elder*" OR "Geriatric Patients" OR "Older Adults" OR "Elderly" OR "Geriatric*" OR senior*)) | 2 |
| (TITLE-ABS-KEY("Primary Health Care" OR "Family Practice" OR "General Practice" OR "Primary care" OR "family medicine")) | 3 |
| **#1 AND #2 AND #3** | 4 |
